# Big on Change, Small on Innovation: Evolutionary Consequences of RNA Sequence Duplication

**DOI:** 10.1007/s00239-019-09906-3

**Published:** 2019-08-21

**Authors:** Andrew Plebanek, Caleb Larnerd, Milena Popović, Chenyu Wei, Andrew Pohorille, Mark A. Ditzler

**Affiliations:** 1 Exobiology Branch, Space Science and Astrobiology Division, NASA Ames Research Center, Bldg N239 Mail Stop 239-4, Moffett Field, CA 94035 USA; 2 Department of Pharmaceutical Chemistry, University of California San Francisco, San Francisco, CA 94143 USA; 3 NASA Internship Program, NASA Ames Research Center, Moffett Field, CA 94035 USA; 4 Center for the Emergence of Life, NASA Ames Research Center, Moffett Field, CA 94035 USA; 5 Blue Marble Space Institute of Science, Seattle, WA 98145 USA

**Keywords:** RNA, Duplication, Aptamer, Structure

## Abstract

The potential for biopolymers to evolve new structures has important consequences for their ability to optimize function and our attempts to reconstruct their evolutionary histories. Prior work with in vitro systems suggests that structural remodeling of RNA is difficult to achieve through the accumulation of point mutations or through recombination events. Sequence duplication may represent an alternative mechanism that can more readily lead to the evolution of new structures. Structural and sequence elements in many RNAs and proteins appear to be the products of duplication events, indicating that this mechanism plays a major role in molecular evolution. Despite the potential significance of this mechanism, little experimental data is available concerning the structural and evolutionary consequences of duplicating biopolymer sequences. To assess the structural consequences of sequence duplication on the evolution of RNA, we mutagenized an RNA sequence containing two copies of an ATP aptamer and subjected the resulting population to multiple in vitro evolution experiments. We identified multiple routes by which duplication, followed by the accumulation of functional point mutations, allowed our populations to sample two entirely different secondary structures. The two structures have no base pairs in common, but both structures contain two copies of the same ATP-binding motif. We do not observe the emergence of any other functional secondary structures beyond these two. Although this result suggests a limited capacity for duplication to support short-term functional innovation, major changes in secondary structure, like the one observed here, should be given careful consideration as they are likely to frustrate attempts to infer deep evolutionary histories of functional RNAs.

**Electronic supplementary material:**

The online version of this article (10.1007/s00239-019-09906-3) contains supplementary material, which is available to authorized users.

## Introduction

Duplication events are a major driver of genetic innovation, and may have played an important role in the early evolution of life. Gene duplications result in redundant copies of a functional sequence, which are released from selective pressure to maintain the structure and function of the parent sequence (Orgel 1977; Nowak et al. 1997; Bergthorsson et al. 2007). Additionally, internal duplication events (i.e. duplication of sequence elements within a gene) can promote the evolution of larger and more complex structures. This evolutionary mechanism has been implicated in the evolution of several important RNA architectures. Modern tRNA structures may have originated from duplication of a hairpin or minihelix RNA, a hypothesis supported by quantitative analysis of tRNA sequences and structures (Widmann et al. 2005; Tanaka and Kikuchi 2001), and by the ability of minihelices to serve as substrates for aminoacyl-tRNA synthetases (Schimmel and Alexander 1998). The structure of the peptidyl transferase center of the ribosome also exhibits symmetry that is suggestive of a duplication event (Agmon et al. 2005; Tamura 2011); however, this apparent symmetry has yet to be described quantitatively. Similar histories of duplication and recombination have also been inferred for various protein architectures, in particular, numerous transmembrane proteins (Shimizu et al. 2004). Additionally, quantitative analyses of TIM barrel architectures indicate that this family of proteins also evolved through internal duplication events (Goldman et al. 2016). The antiquity of these RNA and protein structures suggests that sequence duplication was available as a mechanism in the early evolution of life.

Nucleic acids with duplicated sequence can arise through a variety of mechanisms, such as rolling-circle replication, replication slippage during template-directed synthesis, transposition, or ligation of dimerized RNAs of the same or closely related sequence (Flores et al. 2011; Zhou et al. 2014; Kaessman et al. 2009; Mutschler et al. 2018; Smail et al. 2019). Any or all of these mechanisms could have been accessible during the early history of life and may have served as a means for functional RNAs to grow in size and complexity. Repeated sequence elements contribute to functional architectures in modern RNAs, supporting the utility of duplication during RNA evolution. Examples include GTP aptamers consisting of tandem repeats of short sequence elements (Curtis and Liu 2014), and the Varkud satellite (VS) ribozyme, which forms a functional dimer of two identical sequences generated through rolling-circle replication (Suslov et al. 2015). In addition to its role in naturally occurring RNAs, sequence duplication has been used as a tool in engineered RNAs for biotechnology applications (Zhang et al. 2015).

**Fig. 1:**
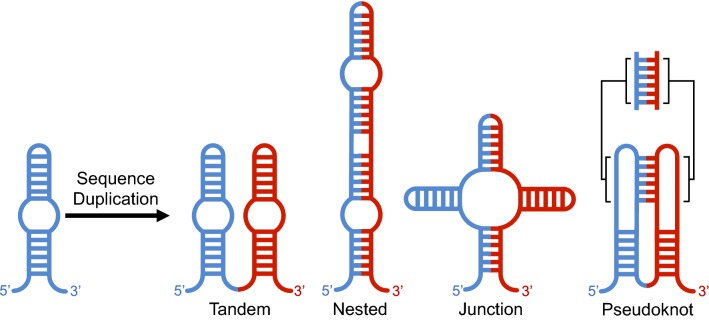
Potential secondary structures arising from the duplication of a generic internal loop. Both the “tandem” and “nested” structures contain two instances of the internal loop found in the original RNA, in the nested form, the internal loop is formed by base-pairing between elements in the two copies of the duplicated sequence. The “junction” and “pseudo-knot” structures disrupt both internal loops

Duplication events have a number of potential consequences for the folding and evolution of RNAs. Duplication of a sequence increases RNA structural plasticity (i.e. the number of available structures of comparable free energy). This is because parts of the sequence that can base-pair within one copy of a duplicated sequence also have the potential to form base pairs between copies (Fig. 1), a feature that is likely to be important in distinguishing RNA duplication from duplication in proteins. RNA duplication can result in multiple secondary structures that retain elements of the original architecture, and thereby maintain or increase the activity of the RNA. Alternatively, duplication can result in structures that disrupt the original functional element and likely (though not necessarily) result in a non-functional structure. The potential to form completely different secondary structures that preserve the original functional element, suggests that sequence duplication may be a potent mechanism for the evolution of novel structures. Earlier studies indicate that other mechanisms are limited in their ability to promote structural changes. In contrast to early theoretical suggestions (Schuster et al. 1994; Huynen 1996), the potential to form new structures through point mutations appears to be limited (Jiménez et al. 2013; Petrie and Joyce 2014; Bendixsen et al. 2017; Pressman et al. 2019). Recombination of different RNA motifs likewise appears to be limited in its ability to support the evolution of new structures (Burke and Willis 1998). The introduction of random sequences adjacent to or within RNA motifs also does not result in the evolution of alternative functional structures (Majerfeld et al. 2010; Popović et al. 2019).

**Fig. 2:**
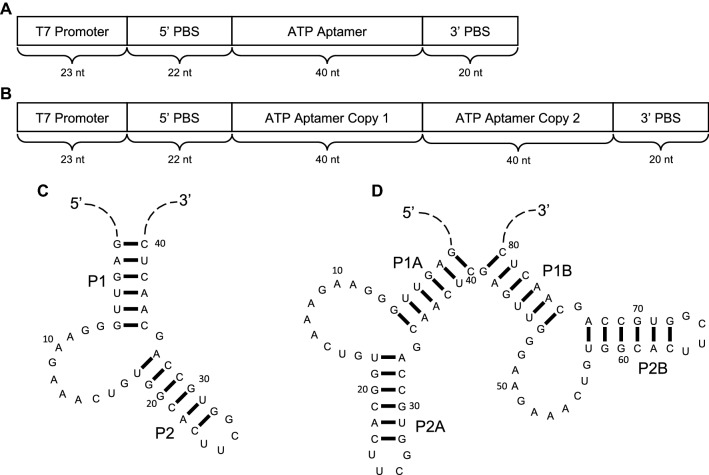
Constructs used in this study. DNA templates contain a T7 promoter sequence, primer-binding sites (PBS) for PCR and reverse transcription, and either one (**a**) or two (**b**) copies of the ATP aptamer sequence. Secondary structures for the single-aptamer (**c**) and double-aptamer (**d**) RNA constructs are shown. In the secondary structures, the sequences subjected to mutagenesis are shown and the 5′ and 3′ constant regions are represented as dashed lines

To observe directly how RNA responds to sequence duplication, we designed and evolved populations of RNAs containing two copies of a well-characterized (Dieckmann et al. 1996, 1997; Burke and Gold 1997), naturally occurring (Vu et al. 2012) adenosine triphosphate (ATP) aptamer (Fig. 2) that was originally identified through in vitro evolution experiments (Sassanfar and Szostak 1993). This ATP aptamer consists of an asymmetric internal loop with one side conforming to the pattern “GGNAGA[N]_2–3_YG” (using IUPAC standard notation with Y denoting pyrimidines and N indicating any nucleotide) and a single conserved “G” on the other side. When these sequence constraints are satisfied, the *K*_d_ for this aptamer is in the low micromolar range (Sassanfar and Szostak 1993; Vu et al. 2012). Small deviations from this sequence pattern frequently result in > 100-fold increases in *K*_d_ (Dieckmann et al. 1997). This aptamer motif recognizes ATP primarily through the nucleobase and it discriminates against other NTPs, including GTP (Sassanfar and Szostak 1993; Dieckmann et al. 1996, 1997 Huang and Szostak 2003). We introduced random mutations into our duplicated ATP aptamer construct (Fig. 2b), and then evolved the resulting populations of mutants to bind ATP. We also attempted to evolve RNAs that are capable of simultaneously binding ATP and GTP. Although our selections for simultaneous ATP/GTP binding resulted in the selection of sequences that, at best, exhibit only limited simultaneous binding activity, they did favor the emergence of a large-scale structural change more strongly than the selections for ATP binding alone. This structural change disrupted all base pairs of the starting construct while retaining two ATP-binding internal loops.

## Results

**Fig. 3:**
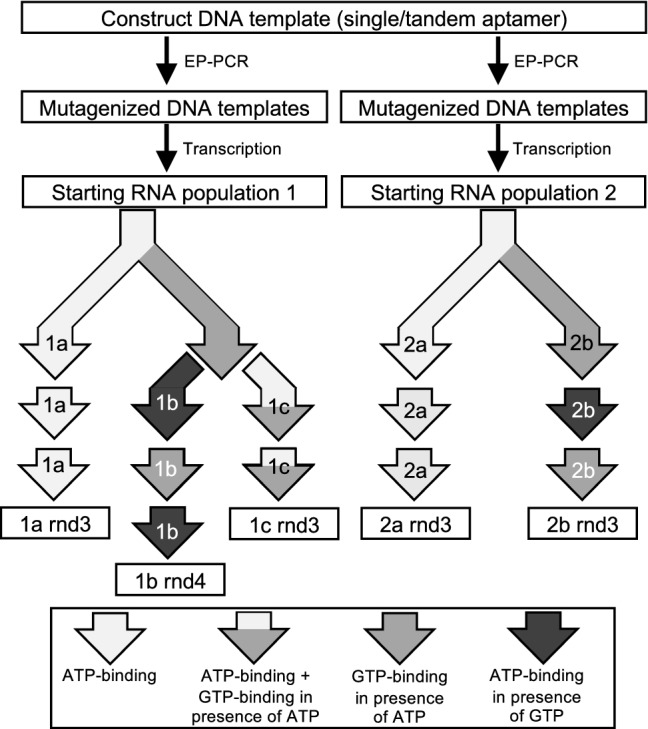
Schematic of in vitro experimental trajectories. Two independently mutagenized populations were generated from both constructs separately and then transcribed in vitro. Starting population 1 was used in a selection for ATP-binding (trajectory 1a) and two dual ATP–GTP-binding selections under differing conditions (trajectory 1b, and 1c). Starting population 2 was used to replicate the first ATP-binding selection (trajectory 2a) and a third dual ATP–GTP-binding selection (trajectory 2b). Shading of arrows is used to indicate the nature of the selection step as indicated in the key at the bottom of the figure

We designed an RNA construct predicted to fold into two functional copies of an ATP aptamer motif (db) and a second construct with a single copy of the aptamer motif (s) to serve as a control (Fig. 2). We used error-prone PCR (EP-PCR) (Cadwell and Joyce 1992) to introduce random mutations to the aptamer sequences of both constructs. In the resulting mutagenized populations, 99% of reads contained mutations in the db populations and 96% of reads in the s populations contained mutations, consistent with error rates per position of 6% and 8%, respectively. We then evolved these populations along multiple experimental trajectories (Fig. 3). Selections for ATP-binding activity were carried out in three rounds of evolution along two trajectories for both the db and s populations. In each round, the populations were incubated with an ATP-functionalized column, and then bound sequences were recovered by adding free ATP for an affinity elution. Selections intended to evolve simultaneous ATP/GTP binding were performed using two different strategies. In the first strategy, the rounds of evolution were alternated between rounds of binding to ATP columns and rounds of binding to GTP columns. In the second strategy, binding to and elution from an ATP-functionalized column was followed immediately by binding to and elution from a GTP-functionalized column in the presence of free ATP, all within a single round of evolution.

**Fig. 4:**
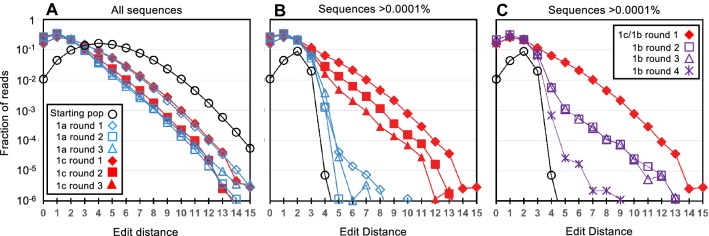
Change in db population structure during evolution. **a** The fraction of sequence reads is plotted as a function of edit distance for the db populations indicated in panel **a**. The data plotted includes sequence reads from all sequences. **b** and **c** The fraction of sequence reads corresponding to sequences that are present as > 0.0001% of the population is plotted as a function of edit distance for the db populations indicated in panel **a** and **c** respectively

As the populations evolved, there was a decrease in the proportion of mutants at high-edit distance (total number of substitutions, insertions, and deletions relative to original sequence) (Fig. 4a). However, for experimental trajectories that included GTP column-binding steps, a subset of high-edit distance sequences increased in abundance within the db populations. This is readily observed by way of comparing the distribution of high-abundance sequences (sequences that are > 0.0001% of the populations) in the db starting population to their distribution within the db populations from trajectory 1c (Fig. 4b). In contrast, rounds of evolution that only require ATP-column binding, strongly select against the high-edit distance sequences (Fig. 4b and c and Supporting Information Figure S1). For example, high-abundance, high-edit distance sequences are not observed in the db populations along trajectories 1a and 2a (Fig. 4b and Supporting Information Figure S1). In addition, when trajectories 1b and 2b (Fig. 3) pass through a round of ATP-column binding, the fraction of high-abundance, high-edit distance sequences decreases more dramatically than it does following the GTP column-binding rounds (Fig. 4c and Supporting Information Figure S1). These observations indicate that the altered selection pressure introduced by GTP column-binding favors specific subsets of high-edit distance mutants in the db populations.

For the s populations, both ATP-binding and simultaneous ATP/GTP-binding selections favor mutants that preserve the secondary structure of the original ATP aptamer. These sequences primarily have compensatory mutations that change the sequence while conserving the original base pairing (Supporting Information Figure S2). There is no indication of alternative structures being favored in any of the trajectories for the s construct. For the s populations, sequences that increase in abundance more than the original unmutated sequence, all contain loops that conform to the sequence pattern GGNAGA[N]_2–3_YG. These results are consistent with prior characterizations of this aptamer and its requirements for ATP binding (Sassanfar and Szostak 1993; Burke and Gold 1997; Vu et al. 2012).

**Fig. 5:**
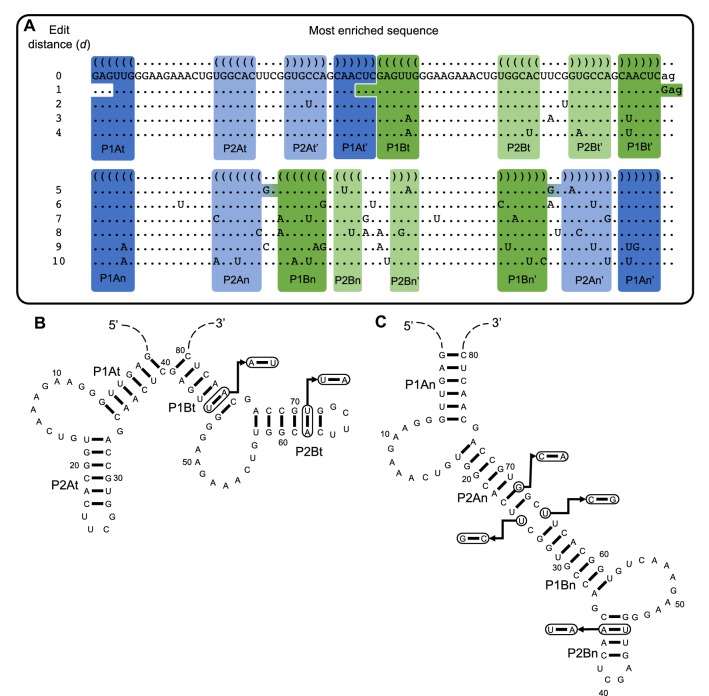
The sequences and structures of the most enriched sequences, at a given edit distance from the “wild-type” sequence, along trajectory 1c. **a** Among sequences with a given edit distance (*d*) the most enriched sequence in the population is shown. Sequences are aligned to the “wild-type” and their corresponding secondary structures. Standard bracket notation is used for secondary structures and pairing regions are highlighted, color-coded, and labeled. For values of *d *= 1–4, the mutations are shown with respect to the tandem secondary structure, and for *d* = 5–10 mutations are shown with respect to the nested secondary structure. **b** An example of mutations in a sequence that is predicted to form the tandem structure (*d *= 4) mapped onto its predicted secondary structure. The sites of the mutations are circled within the structure and arrows are used to indicate the altered sequence and any new base pairs formed. **c** An example of mutations in a sequence predicted to form the nested structure (*d *= 5) mapped onto the nested secondary structure

**Fig. 6:**
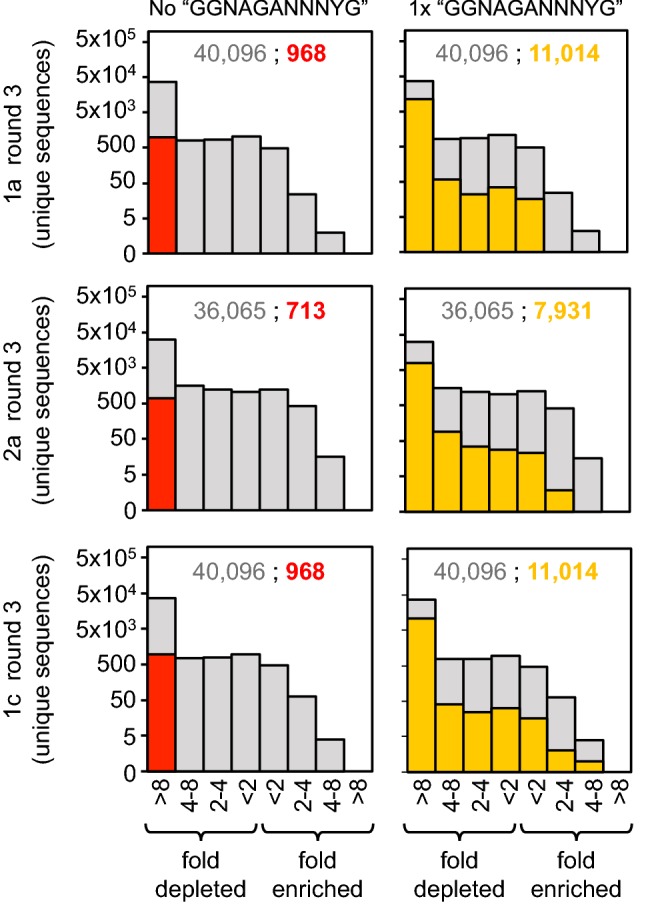
Histograms of enrichment or depletion of sequences present at an abundance of ≥ 0.0001% in the starting population data set for the db construct. Enrichment values are based on the change in the number of reads relative to the original “wild-type” sequence. Histograms in both columns show the enrichment/depletion of all sequences in the starting population as light gray bars. For histograms on the left, sequences containing no instances of the pattern “GGNAGA[N]_2–3_YG” are shown with red bars. For histograms on the right, sequences containing only one instance of this pattern are shown in yellow. Numbers given as inset are the total number of unique enriched/depleted sequences in light gray followed by the number of unique sequences corresponding to the indicated subset red and yellow (Color figure online)

**Fig. 7:**
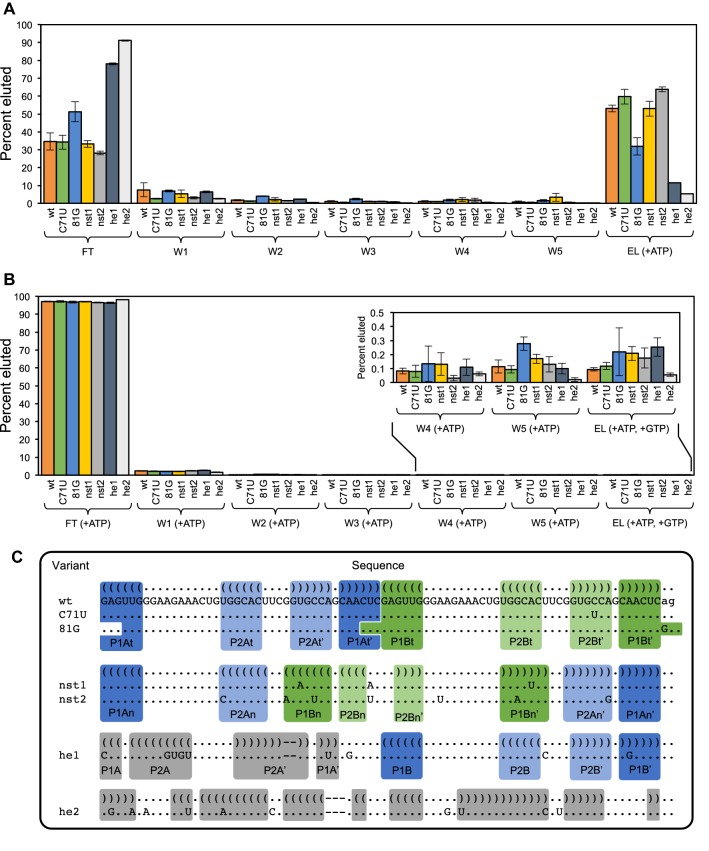
Competitive binding assays for selected sequences. Binding to and elution from **a** ATP-agarose columns and **b** GTP-agarose columns in the presence of 1 μM competitor “wild-type” db aptamer. The percent of ^32^P labeled RNA detected in each fraction (flow through, washes W1–W5, and elution) are plotted **c** An alignment of the sequences is shown below. Pairing regions that support formation of the original ATP aptamer loop are highlighted in blue or green. Pairing regions that do not support formation of the original ATP aptamer motif are highlighted in gray (Color figure online)

Similar to the results for the s populations, in the db populations, among the mutants with low-edit distances (*d* ≤ 4), those that are most enriched are predicted to form the same tandem secondary structure as the original “wild-type” sequence (Figs. 2d, 5a, b, and Supporting Information Figure S3). These sequences have mutations that maintain the tandem structure while disrupting base pairs in competing structures. The db populations also maintain the original GGNAGA[N]_2–3_YG sequence pattern. Sequences observed in the starting population that do not contain this sequence pattern are depleted along all experimental trajectories, and nearly all RNAs containing only one copy of the sequence pattern are also depleted (Fig. 6). Additionally, RNAs that do not have two copies of the GGNAGA[N]_2–3_YG sequence or destabilize the formation of one or both binding loops are outcompeted by the “wild-type” sequence in individual, competitive binding assays (Fig. 7). Two mutants at high-edit distance, designated he1 (*d* = 11) and he2 (*d* = 13), contain only one copy or no copies of this sequence pattern respectively (Fig. 7c) and exhibit very limited binding to the ATP-functionalized column in the presence of “wild-type” competitor (Fig. 7a). 81G, a mutant that partially destabilizes one copy of the aptamer loop, displays higher affinity for the ATP column than he1 and he2, but does not bind as well as the wild-type (Fig. 7a and c).

**Fig. 8:**
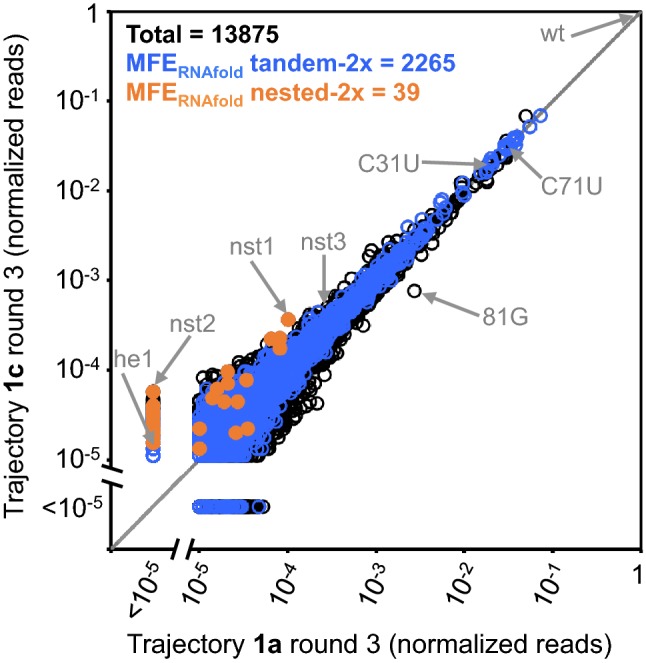
Relative abundance of sequences present in populations 1a round-3 and 1c round-3. All sequences whose abundance is greater than 0.001% of the “wild-type” in either population are plotted. Points above the diagonal represent sequences with higher relative abundance in trajectory 1c round-3 and points below have higher abundance in trajectory 1a round-3. Sequences with predicted minimum free energy structures that form two copies of the aptamer loop in either the tandem (blue) are nested (orange) conformation are indicated, and the number of sequences predicted to form these minimum free energy structures are given at the top of the graph. Specific sequences mentioned in the text are indicated with arrows (Color figure online)

In the db populations, several mutants are predicted to form an alternative secondary structure. These mutants, which are especially prevalent among sequences at high-edit distances (*d* ≥ 5) (Fig. 5a), are predicted to adopt a nested secondary structure (Fig. 1) that has no base pairs in common with the tandem secondary structure (Fig. 5c). Yet, the nested secondary structure still contains two copies of the conserved ATP-binding loop (Fig. 5a and c and Supporting Information Figure S3). These sequences form the nested secondary structure by destabilizing stems that support the tandem structure, and in some cases they support the formation of additional base pairs that increase the stability of the nested stems. For example, the most-enriched sequence at edit distance *d* = 5 along trajectory 1c (Fig. 3) has mutations that are disruptive to the tandem structure but in the nested arrangement allow for formation of an uninterrupted 16 base-pair stem between the two copies of the conserved ATP-binding loops (Fig. 5a and c). Although most of the sequences predicted to form the nested structure are at high-edit distances, several are also present at low-edit distances. For example, the sequences nst1 and nst3 only differ from the original “wild-type” sequence by 3 and 2 edits, respectively. Notably, mutants predicted to form the nested structure, regardless of edit distance, are more abundant in the populations evolved with the dual ATP/GTP selections than they are in the ATP-only selections (Fig. 8). However, our competitive binding assays indicate that the nested conformation supports only limited, if any, dual ATP/GTP binding (Fig. 7b).

**Fig. 9:**
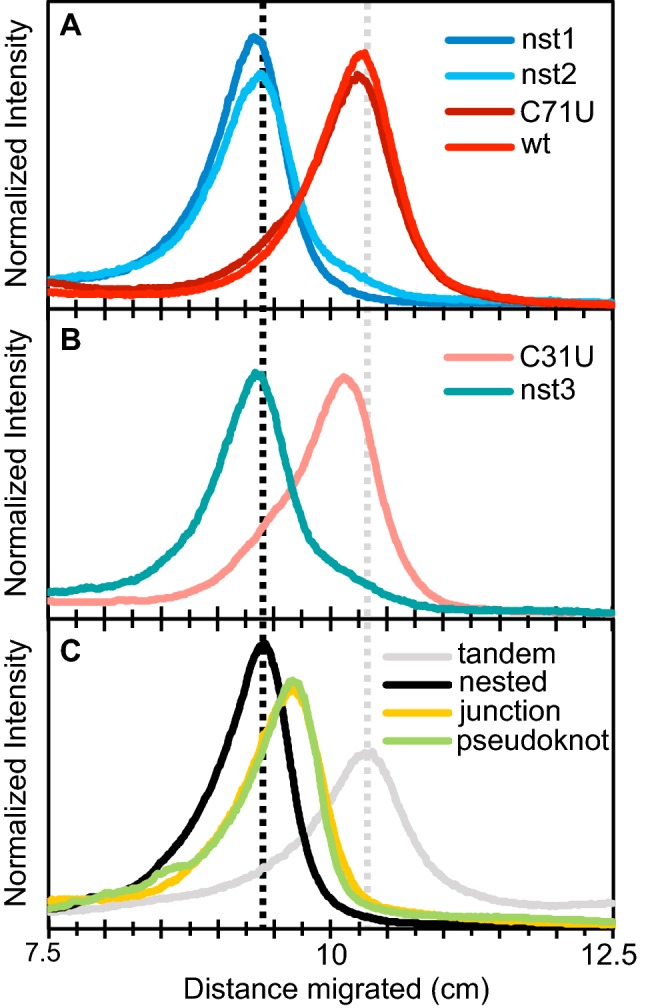
Electropherograms from native PAGE mobility assays. **a**, **b** Mobility of sequences from the evolved populations as indicated in the key within each plot. Sequences predicted to form the tandem structure migrate faster than sequences predicted to form the nested structure. **b** Two sequences that differ by a single point mutation, and are predicted to favor two different structures, have correspondingly different mobilities. **c** Sequences selected to act as structure standards. The position of the peaks of the electropherograms for the tandem and nested structures are indicated in all three panels with gray and black-dashed lines, respectively

Consistent with their predicted structures, sequences predicted to fold into the nested structure have a characteristic electrophoretic mobility in native gels that is distinct from the mobility of sequences predicted to fold into the tandem configuration (Fig. 9a, b). The mobility of a reference sequence designed to fold into the nested structure, and the mobility of a reference sequence predicted to form the tandem structure with high probability further confirm the accuracy of the secondary structure predictions (Fig. 9c). Sequences designed to form a “junction” or a “pseudo-knot” secondary structure, comparable to those shown in Fig. 1, which do not form the original ATP-binding loop, have an intermediate mobility distinct from that observed for any of the evolved sequences assayed.

**Fig. 10:**
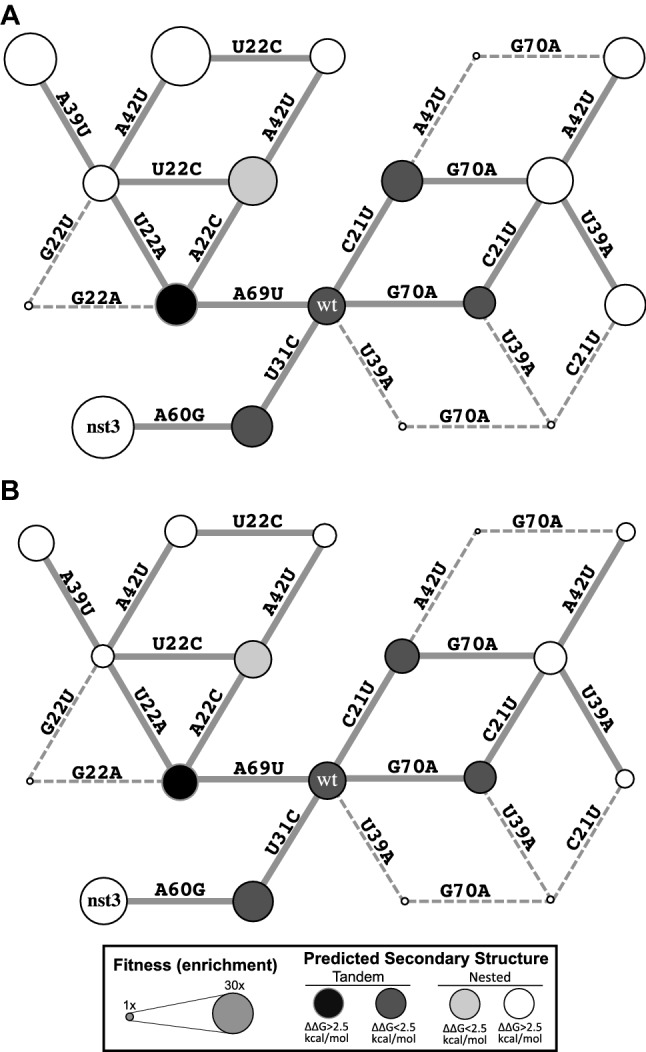
A network of mutations connects tandem and nested structures in sequence space. Sequences are represented by circles and lines connecting them represent single point mutations that convert one sequence to another. Connections to fit sequences are shown as solid lines and connections to sequences that perform poorly in the evolution experiment are shown as dashed lines. The size of the circles is proportional to the enrichment of the sequence along **a** trajectory 1c or **b** trajectory 1a. The shading of the circles indicates both whether the tandem (black and dark gray) or nested (white or light gray) structure is predicted to be the more stable of the two conformations and the magnitude of the predicted free energy difference between the ensemble of tandem and nested structures. Shading indicates whether the magnitude of the free energy difference is greater than (white and black) or less than (light gray and dark gray) 2.5 kcal/mol

Within the evolved populations, evolutionary paths can be constructed in sequence space that connect sequences that fold into the tandem secondary structure to those that fold into the nested secondary structure through a continuous series of point mutations between evolutionarily fit sequences (Fig. 10 and Supporting Information Figure S4). Figure 10 illustrates a series of point mutations that can convert the “wild-type” sequence (tandem structure, faster gel mobility) into different, highly fit sequences that form the nested structure. These examples are two to three edits away from the wild-type, and therefore, reside within a region of sequence space that is well sampled in our starting populations (Fig. 4). The “wild-type” sequence can evolve to nested structures through a series of increasingly fit mutants. For example, the “wild-type” can evolve to nst3 (*d* = 2) through two successive mutations, both of which result in increased fitness along both ATP/GTP (Fig. 10a) and ATP only (Fig. 10b) experimental trajectories. Each of these paths connecting the “wild-type” to close (*d* ≤ 3) nested sequences passes through one of five specific point mutants (four of which are illustrated in Fig. 10) whose minimum free energy structure is predicted to be tandem. This suggests that a specific, limited set of point mutations to the wild-type sequence predispose the RNA to transition to the new secondary structure and the vast majority of the 240 possible point mutants decrease the probability of a transition. Additionally, each of these paths includes mutation to the stems P2At and P2Bt (Fig. 2), indicating a preferred mechanism for evolution of the new structure. No path was found connecting “wild-type” to nst2 or to any other nested sequence at edit distance > 3. However, these sequences reside within a region of sequence space that is poorly sampled in the populations. This means that our inability to identify a path may simply reflect limited sampling rather than an actual absence of a viable evolutionary path.

Along the evolutionary paths that connect tandem and nested structures, the computationally predicted minimum free energy structures pass directly from tandem to nested in a single step, but for many of these sequences both tandem and nested conformations are likely significantly sampled within the ensemble of low energy structures. Along most paths connecting sequences with a tandem minimum free energy structure to sequences with a nested minimum free energy structure, the magnitude of the predicted free energy difference between tandem and nested structures decreases and then increases as the predicted ensemble of secondary structures shifts progressively from one set of conformations to the other (Supporting Information Figures S5 and S6). For sequences that are just one step away from shifting between tandem and nested minimum free energy structures, native gel mobility assays provide evidence that multiple structures are significantly populated within their respective ensembles. The sequences C31U and nst3 are only one step away from each other and the electropherograms for both of these sequences have more pronounced shoulders (Fig. 9b) than the other sequences tested (Fig. 9a). These observations indicate that prior to a major shift in the minimum free energy structure the new structure becomes increasingly populated within the ensemble of low energy structures.

## Discussion

Sequence duplication presents an evolving RNA population with both challenges and opportunities. Duplication increases the number of non-functional confirmations, but also provides a mechanism to change secondary structure while maintaining or enhancing function. Duplication of any functional internal loop, such as the one in the ATP aptamer examined here, leads to at least four possible alternative secondary structures that contain a similar number of base pairs (Fig. 1) and exhibit comparable predicted free-energy values. In our system, we observe that sequence duplication affords closely related sequences multiple opportunities to evolve between radically different secondary structures (Fig. 10) that have no base pairs in common (Fig. 5b, c). Our structure predications are supported by native gel mobility assays (Fig. 9) and our activity assays confirm that both tandem and nested structures have comparable binding activity (Fig. 7).

Prior in vitro evolution experiments have shown that several evolutionary mechanisms are inefficient at accessing novel structures. The evolution of new structures through point mutations alone has proven challenging, as evidenced by many in vitro evolution studies. Mutagenesis of in vitro evolved RNAs, followed by reselection for the original function, reliably results in the evolution of multiple sequence variants that conform to the original RNA structure (Pitt and Ferre-D’Amare 2010; Petrie and Joyce 2014; Bendixsen et al. 2017). And in vitro evolution experiments that exhaustively map the fitness of all possible sequences for very short RNAs, reveal disconnected networks of functional sequences that cannot be connected by continuous paths of fit mutations (Jiménez et al. 2013; Pressman et al. 2019). In at least one system, continuous paths of fit mutations connecting two different structures have been identified (Schultes and Bartel 2000; Bendixsen et al. 2019). However, because these paths were discovered between sequences that were specifically designed by the experimenter to be connected through a continuous path of fit point mutations (Schultes and Bartel 2000) and the subsequent exploration of paths between them was highly restricted (Bendixsen et al. 2019), it is unclear how likely it is that freely evolving RNA population would follow such paths.

In addition to point mutations, neither recombination of separately evolved structures, nor the introduction of random flanking sequences appears to favor the evolution of new structures. When aptamers that bind either coenzyme A, chloramphenicol, or adenosine, are recombined, mutagenized, and then reselected for the original binding functions, the resulting RNA population contain sequences that fold into the original structures (Burke and Willis 1998). In addition, when the conserved core of a tryptophan aptamer is inserted into arbitrary sequences with 14 to 18 fully randomized positions, and then selected for tryptophan binding, the resulting sequences form the same functional core (Majerfeld et al. 2010). Finally, our own recent observations reveal that the introduction of random sequences within or adjacent to the conserved core structure of an in vitro evolved ligase ribozyme does not enable the evolution of a new core structure (Popović et al. 2019). In contrast to the studies described above, we observe multiple opportunities to evolve between two completely different secondary structures. It would therefore appear that sequence duplication occupies a privileged position among evolutionary mechanisms in its ability to support the emergence of a new secondary structure. However, we only observe evidence of these two functional structures emerging in the population, even when the selection pressure was altered to include a GTP-binding step. This suggests that even following duplication the number of accessible functional structures, and potential for functional innovation remains limited.

In spite of the redundancy of the ATP-binding loop, we do not see evidence of aptamers with pronounced dual-binding activity emerging. In prior in vitro evolution experiments, GTP aptamers were derived from the same ATP aptamer sequence used in our study. The derived GTP aptamers were at high-edit distances (*d* = 7–12) within the single copy (Huang and Szostak 2003) and did not require retention of ATP affinity. The lower level of mutagenesis employed here, may explain why one copy of the ATP-binding loop did not evolve into a high-affinity GTP-binding structures that can support clear, dual ATP/GTP binding. The absence of any high-affinity GTP-binding structures in our less heavily mutagenized populations suggests that continuous evolutionary paths of functional point mutants leading to GTP aptamers may be extremely rare or non-existent. Similarly, another known RNA structure that has both high affinity for ATP and lower but clearly detectable affinity for GTP (Sazani et al. 2004), does not emerge in our populations, suggesting a scarcity of paths to alternative ATP-binding structures as well.

In our evolution experiments, different selection pressures result in a small but clear preferential enrichment of different structures, with the ATP-binding trajectories favoring the tandem secondary structures, whereas ATP/GTP-binding trajectories favor nested secondary structures. Although RNAs with nested secondary structures are favored in ATP/GTP-binding selections (Fig. 8), our binding assays are only consistent with a very limited ability for the nested structures to bind both ATP and GTP simultaneously (Fig. 7b). The preferential enrichment of nested sequences in ATP/GTP-binding selections may even be a result of non-specific binding, or other factors unrelated to ATP/GTP binding. Regardless of the mechanism responsible, it is nonetheless clear that the selection pressures of the ATP/GTP selections favor a population in which alternative secondary structures are more evenly represented. This observation is consistent with prior evidence for decanalization in response to changes in selection pressure during in vitro evolution (Hayden et al. 2012), and it demonstrates how a small selective advantage can still have a significant impact on the emergence of new structures.

Examining the network of sequences at the interface of those that support either the tandem structure or the nested structure reveals details about how a structural transitions can proceed, though as discussed above, this is likely a rare occurrence. Theoretical studies of RNA evolution, based on simulated RNA folding, have highlighted the potential of RNAs to evolve from sequences for which a given structure is only a minor component of its ensemble of conformations to a sequence for which that structure is the most populated conformation in the ensemble (Fontana and Schuster 1998; Ancel and Fontana 2000). We observe such a transition here, with the nested structure appearing as a minor component of the “wild-type’s” predicted ensemble of stable conformations, and then becoming the major component for sequences only a couple of steps away in sequence space. Consistent with theoretical work, this transition appears to pass through intermediates that significantly sample both functional conformations and only a small fraction of the “wild-type’s” point mutations support this transition. A similar transition, that did not involve duplication, was recently reported for the evolution of new protein structures during phage evolution in which bistable sequences appeared along the path between two distinct structures (Petrie et al. 2018). Unlike the behavior observed in theoretical models, our populations do not appear to have access to a wide variety of functional structures. Rather, they are only able to access the one additional functional structure whose presence in the “wild-type’s” conformational ensemble is a nearly inevitable consequence of sequence duplication. Although the emergence of this additional structure represents a large structural change, both structures undoubtedly bind ATP through the same mechanism. Additionally, the functional change that provides a selective advantage to the nested structure in the GTP column-binding step is small, with only a fraction of a percent of the RNA being recovered in the elution. This system, therefore, demonstrates how a very small functional innovation can promote the evolution of a very large structural change as a consequence of sequence duplication.

Finally, our observations have important implications for attempts to infer the evolutionary history of naturally occurring RNAs (Tanaka and Kikuchi 2001; Mandal et al. 2004; Agmon et al. 2005; Widmann et al. 2005; Tamura 2011; Petrov et al. 2014). Duplication followed by rearrangement of the secondary structure is a mechanism that was previously proposed in models of tRNA evolution (Widmann et al. 2005; Tanaka and Kikuchi 2001), and our results support the plausibility of this mechanism. Our results also demonstrate duplication followed by reinforcement of the original secondary structure, which appears to have occurred in some riboswitches (Mandal et al. 2004). Significantly, our results highlight challenges in uncovering the deep evolutionary history of RNAs and the limitations of relying on secondary structures as a guide in this endeavor. Duplication events followed by global structural changes, similar to those observed here (Figs. 1 and 5), may become indistinguishable from insertion events when observed after the fact, especially after the accumulation of additional sequence changes. For example, our nested structures, which arose from sequence duplication, could be mistakenly interpreted as the result of inserting a second copy of the aptamer in the middle of the first. Additionally, if one of the copies of the functional motif evolves into a new structure, the duplication event may eventually become entirely indistinguishable from the insertion of an unrelated sequence. It is, therefore, important to consider duplication followed by structural rearrangements as an alternative to insertion events when reconstructing the evolutionary history of functional RNAs.

## Methods

### Generation of Mutagenized RNA Populations

DNA oligonucleotides were synthesized by Integrated DNA Technologies. Oligos were heat annealed, and extended using DNA polymerase I Klenow fragment (Thermo Scientific) to yield the full construct DNA templates. We used an error-prone polymerase chain reaction (EP-PCR) protocol to introduce random mutations to the variable regions of both s and db DNA templates (Cadwell and Joyce 1992). The mutagenized DNA libraries were transcribed using T7 RNA Polymerase to yield populations of RNA molecules. RNA was purified using denaturing polyacrylamide gel electrophoresis (PAGE), eluted from gel fragments using 300 mM sodium acetate, pH 5.3, ethanol precipitated, and resuspended in deionized water.

### In Vitro Evolution Procedure

Selections for ATP-binding activity were carried out via affinity chromatography. Prior to selection, 3 × 10^14^ molecules of RNA were suspended in 1 mL of buffer 3-(*N*-morpholino)propanesulfonic acid (MOPS) pH 7.5, 5 mM MgCl_2_, 200 mM NaCl and heated to 70 °C for 60 s. After allowing 15 min for cooling, RNA samples in buffer were added to affinity columns containing 10 mg of ATP-agarose beads (Sigma-Aldrich). The ATP-agarose comprised agarose linked to the C8 position of the ATP nucleobase by a 9-carbon chain; 10 mg of ATP-agarose was estimated to contain approximately 6 × 10^13^–3 × 10^14^ ATP molecules available for binding. Columns were tumbled for 1 h at ~ 23 °C to allow binding, after which the flow-through fraction was collected by centrifuging at 2000×*g* for 30 s. Columns were washed by adding 500 μL of MOPS buffer with 5 mM MgCl_2_ and 200 mM NaCl, tumbling for 5 min, and centrifuging. After a total of five washes, ATP-binding RNAs were eluted by adding 200 μL of MOPS buffer with 7 mM MgCl_2_, 200 mM NaCl and 2 mM ATP, tumbling overnight, and centrifuged. To generate the RNA population for subsequent rounds of ATP-binding selection, a 100 μL aliquot of the elution fraction was ethanol precipitated, resuspended, reverse-transcribed using Improm-II reverse-transcriptase (Promega), amplified via PCR using *taq* DNA polymerase, and transcribed using T7 RNA polymerase. Additional variation was introduced by mutations that occur during these amplification steps, but the mutation rate is low (approximate error rates per base pair per duplication for *Taq* DNA polymerase and T7 RNA polymerase are in the range of 10^−4^–10^−5^) and most of the diversification occurred during the initial mutagenic PCR step. The resulting RNA library was then gel-purified, precipitated, and resuspended. The ATP-binding selection process was then repeated using 3 × 10^14^ molecules from the newly generated RNA library.

For ATP/GTP-binding selection along trajectory 1c, 100 μL of the elution from the ATP selection was added directly to a column containing approximately 100 μL of GTP-agarose beads (Sigma-Aldrich). Unlike ATP-agarose, GTP-agarose consists of agarose linked to both 2′ and 3′ hydroxyls of the GTP ribose by 9-carbon chains. The heat-denaturing and refolding step was omitted from this selection to allow ATP-binding mutants to remain bound to ATP in solution during the GTP-binding step. As with the ATP-binding selection, the column was tumbled for 1 h before collection of the flow-through. The column was then washed five times using 500 μL MOPS buffer with 7 mM MgCl_2_, 200 mM NaCl, and 2 mM ATP, followed by overnight elution by tumbling with 200 μL MOPS buffer with 9 mM MgCl_2_, 200 mM NaCl, 2 mM ATP, and 2 mM GTP. The elution fraction was used to generate a new RNA library, where 3 × 10^14^ molecules were used in the subsequent rounds of the dual-binding selection process. For trajectories 1b and 2b, 3 × 10^14^ molecules from the library were added to a column containing ATP-agarose beads with 2 mM GTP in solution and let tumble for 1 h. The flow-through and 5 washes were collected before the column eluted overnight. An RNA library was generated from the elution, then 3 × 10^14^ molecules were run on a column containing GTP-agarose beads with free ATP in solution. A third round of selection followed with another column containing ATP-agarose and GTP in solution.

### Sequence Analysis of Evolved Populations

RNA libraries were prepared for high-throughput sequencing using PCR with primers designed to add index sequences that allow multiplexing of multiple populations on a single sequencing lane. The prepared DNA libraries were sequenced on an Illumina 4000 HiSeq instrument. Only sequence that matched perfectly in both directions of the two paired-end reads were used for analysis. The FASTAptamer toolkit was used to count reads for all sequences, compare abundance of sequences between populations, and determine the edit distances from the original constructs.

### Binding Assays

Binding assays were done under the same conditions as the selection step of our in vitro evolution experiments. Specific mutants (Supporting Information Table S1) were transcribed in vitro and 5′-end labeled with ^32^P. After labeling, samples were passed serially through two size exclusion columns and then precipitated in 300 mM NaOAc, 70% ethanol. Radiolabeled samples were resuspended in binding buffer (50 mM MOPS pH 7.5, 5 mM MgCl_2_, 200 mM NaCl), heated to 70 °C for 60 s, and then cooled for 15 min. The mutants were diluted to 10 nM in binding buffer containing 1 μM “wild-type” sequence, added to columns with 10 mg of ATP-agarose and allowed to bind for 1 h. The columns were then washed five times with binding buffer. Samples were eluted overnight with an ATP containing elution buffer (2 mM ATP, 50 mM MOPS pH 7.5, 7 mM MgCl_2_, 200 mM NaCl). Samples were also run on columns with 100 μL of GTP-agarose using the same protocol. Material from each fraction was quantified through scintillation counting. All binding assays were carried out in triplicate.

### Electrophoretic Mobility Shift Assays

For electrophoretic mobility shift assays (EMSA), specific RNAs (Supporting Information Table S1) were labeled with ^32^P, heat-denatured and cooled in running buffer, then loaded with 10% glycerol onto 6% polyacrylamide gels with running buffer composed of 50 mM tris–acetate, 5 mM magnesium acetate, and 200 mM sodium acetate (pH 7.5). EMSA gels were run at 15 W in thirteen 45-min intervals for a total of 9.75 h of runtime; between intervals, the top and bottom buffer reservoirs were exchanged with fresh buffer to limit formation of a pH gradient between reservoirs. The temperature of the gels and buffer was maintained at ~ 10–15 °C by placing heat-diffusion plates in contact with cooling packs on either side of the gel assembly, the gel and buffer were cooled to 4 °C prior to the run. Gels were exposed overnight to phosphor screens and scanned on a Typhoon phosphorimager. Electropherograms were generated with ImageQuant software.

### Structure Predictions

RNAfold (ViennaRNA2.3.3) (Lorenz et al. 2011) was used to predict the secondary structures of RNA sequences. For an RNA sequence, the ensemble of all structures predicted to be within 2.5 kcal/mol of the minimum free-energy structure were identified. For values used in Fig. 10, predicted structures were considered to be tandem if two aptamer loops were formed by the closing base pairs between positions 6 and 35, 18 and 33, 46 and 75, 58 and 73 within the 80 nt mutagenized region. Structures were considered to be nested if two aptamer loops were formed by the closing base pairs between positions 6 and 75, 18 and 73, 35 and 46, 33 and 58 within the 80 nt mutagenized region. The fraction of the population of suboptimal structures for a sequence *i* in either tandem or nested structure was estimated according to Boltzmann distribution as ${{\sum\limits_{i,s} {{\text{e}}^{{ - \frac{{\Delta G_{i,s} }}{{k_{\text{B}} T}}}} } } \mathord{\left/ {\vphantom {{\sum\limits_{i,s} {{\text{e}}^{{ - \frac{{\Delta G_{i,s} }}{{k_{\text{B}} T}}}} } } {\sum\limits_{{i,{\text{all}}}} {{\text{e}}^{{ - \frac{{\Delta G_{i} }}{{k_{\text{B}} T}}}} } }}} \right. \kern-0pt} {\sum\limits_{{i,{\text{all}}}} {{\text{e}}^{{ - \frac{{\Delta G_{i} }}{{k_{\text{B}} T}}}} } }}$, where Δ*G* is the free energy, *k*_B_ is the Boltzmann constant, *T* is the temperature, *s* is the index for a suboptimal secondary structure defined as tandem or nested structure, and (*i*, all) counts all suboptimal structures included in the analysis, as defined above.

### Shortest Evolution Path Calculations

A connection map for the sequences was generated, on which each sequence was considered as a vertex and if and only if two sequences were related by a single point mutation (including deletion and insertion) the corresponding two vertices were considered connected by an edge (with an equal weighted value). The connection map was calculated for all sequences obtained from the high-throughput sequencing for the in vitro evolution experiments after the third round of selection. The shortest evolution path between any two chosen sequences on the map was identified using the breadth first search (BFS) algorithm (Cormen et al. 2001). The sequences included in the path search were within the highest possible enrichment cutoff to have a connecting path between the two chosen sequences.

## Electronic supplementary material

Below is the link to the electronic supplementary material.

Supplementary material 1 (PDF 558 kb)
