# Supplementary material for: Big on Change, Small on Innovation: Evolutionary Consequences of RNA Sequence Duplication
Source: J Mol Evol. 2019 Aug 21;87(7):240–53. doi: 10.1007/s00239-019-09906-3 (PMC6711949; doi:10.1007/s00239-019-09906-3)
Supplement: Supplementary file 1 — Supplementary material 1 (PDF 558 kb) [file 239_2019_9906_MOESM1_ESM.pdf]

Name	**Sequence** (constant sequence is in lowercase, mutagenized aptamer sequence is in UPPER case)	**Assays run on individual sequence**	**Predicted minimum free energy structure**
wt	gggcgaauaccuuaugcgcacaGAGUUGGGAAGAAACUGUG<br>GCACUUCGGUGCCAGCAACUCGAGUUGGGAAGAAACUGUGG<br>CACUUCGGUGCCAGCAACUCagaucggaagagcgucgugu	Binding, and EMSA	Tandem
C71U	gggcgaauaccuuaugcgcacaGAGUUGGGAAGAAACUGUG<br>GCACUUCGGUGCCAGCAACUCGAGUUGGGAAGAAACUGUGG<br>CACUUCGGUG**U**CAGCAACUCagaucggaagagcgucgugu	Binding, and EMSA	Tandem
nst1	gggcgaauaccuuaugcgcacaGAGUUGGGAAGAAACUGUG<br>GCACUUCGG**A**GCCAGCAAC**A**CGAGUUGGGAAGAAACUGUGG<br>C**U**CUUCGGUGCCAGCAACUCagaucggaagagcgucgugu	Binding, and EMSA	Nested
nst2	gggcgaauaccuuaugcgcacaGAGUTGGGAAGAAACUG**C**G<br>GCACTUC**A**GTG**U**CAGCAAC**G**CGAGUUGGG**U**AGAAACTGUG**A**<br>CACTUCGGTGCC**G**GCAACUCagaucggaagagcgucgugu	Binding, and EMSA	Nested
81G	gggcgaauaccuuaugcgcacaGAGUUGGGAAGAAACUGUG<br>GCACUUCGGUGCCAGCAACUCGAGUUGGGAAGAAACUGUGG<br>CACUUCGGUGCCAGCAACUC**G**agaucggaagagcgucgugu	Binding	Other, see Fig. 7
he1	gggcgaauaccuuaugcgcaca**C**AGUUGGGA**GUGU**ACUGUG<br>GCACUUCUGCC**U**GC**G**ACUCGAGUUGGGAAGAAACUGUGGCA<br>C**C**UCGGUGCC**G**GCAACUCagaucggaagagcgucgugu	Binding	Other, see Fig. 7
he2	gggcgaauaccuuaugcgcacaG**G**GU**A**G**A**GAAGA**U**ACUG**A**G<br>GCACU**C**CGGUGCCAACUCGAGUUGGGA**G**G**U**AACUGUGGCAC<br>**C**U**U**GGUGCCAGCAACUCagaucggaagagcgucgugu	Binding	Other, see Fig.7
nst3	gggcgaauaccuuaugcgcacaGAGUUGGGAAGAAACUGUG<br>GCACUUCGGUG**U**CAGCAACUCGAGUUGGGAAGAAACUGUG**A**<br>CACUUCGGUGCCAGCAACUCagaucggaagagcgucgugu	EMSA	Nested
C31U	gggcgaauaccuuaugcgcacaGAGUUGGGAAGAAACUGUG<br>GCACUUCGGUG**U**CAGCAACUCGAGUUGGGAAGAAACUGUGG<br>CACUUCGGUGCCAGCAACUCagaucggaagagcgucgugu	EMSA	Tandem
Tandem standard	gggcgaauaccuuaugcgcacaGAGUUGGGAAGAAACUGUG<br>GCACUUCGGUGCCAGCAACUCGAGU**G**GGGAAGAAACUGUGG<br>CACUUCGGUGCCAGC**C**ACUCagaucggaagagcgucgugu	EMSA	Tandem
Nested standard	gggcgaauaccuuaugcgcacaGA**CA**UGGGAAGAAACUGUG<br>**C**C**U**CU**GG**GG**A**G**G**CAGC**GC**CU**AG**AG**GC**GGGAAGAAACUGUG**C**<br>C**U**C**CCC**GG**A**G**G**CAGCA**UG**UCagaucggaagagcgucgugu	EMSA	Nested
Junction standard	gggcgaauaccuuaugcgcacaGA**CA**UGGGAAGAAACUGUG<br>**AG**AC**G**UCGGU**CU**CAGC**GC**CU**AG**AG**GC**GGGAAGAAACUGUGG<br>CAC**G**UCGGUGCCAGC**AUG**UCagaucggaagagcgucgugu	EMSA	Other
Pseudoknot standard	gggcgaauaccuuaugcgcacaGA**CA**UGGGAAGAAACUGUG<br>**AG**AC**G**UCGG**A**G**G**CAGCA**UG**UCGAGUUGGGAAGAAACUGUG**C**<br>C**U**C**G**UCGGU**CU**CAGCAACUCagaucggaagagcgucgugu	EMSA	Other

Table S1. Full-length RNA sequences that were subjected to separate individual assays. Mutations relative to the "wt" sequence are in bold.

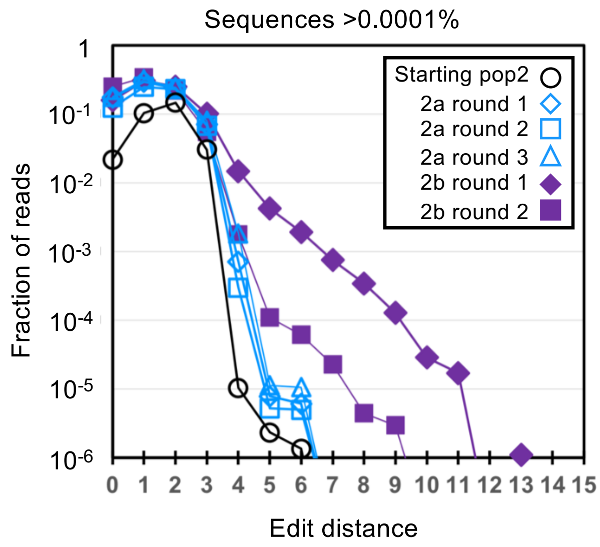

Fig. S1 Change in db population structure during evolution. The fraction of sequence reads corresponding to sequences that are present as >0.0001% of the population is plotted as a function of edit distance for the db populations indicated in the figure.

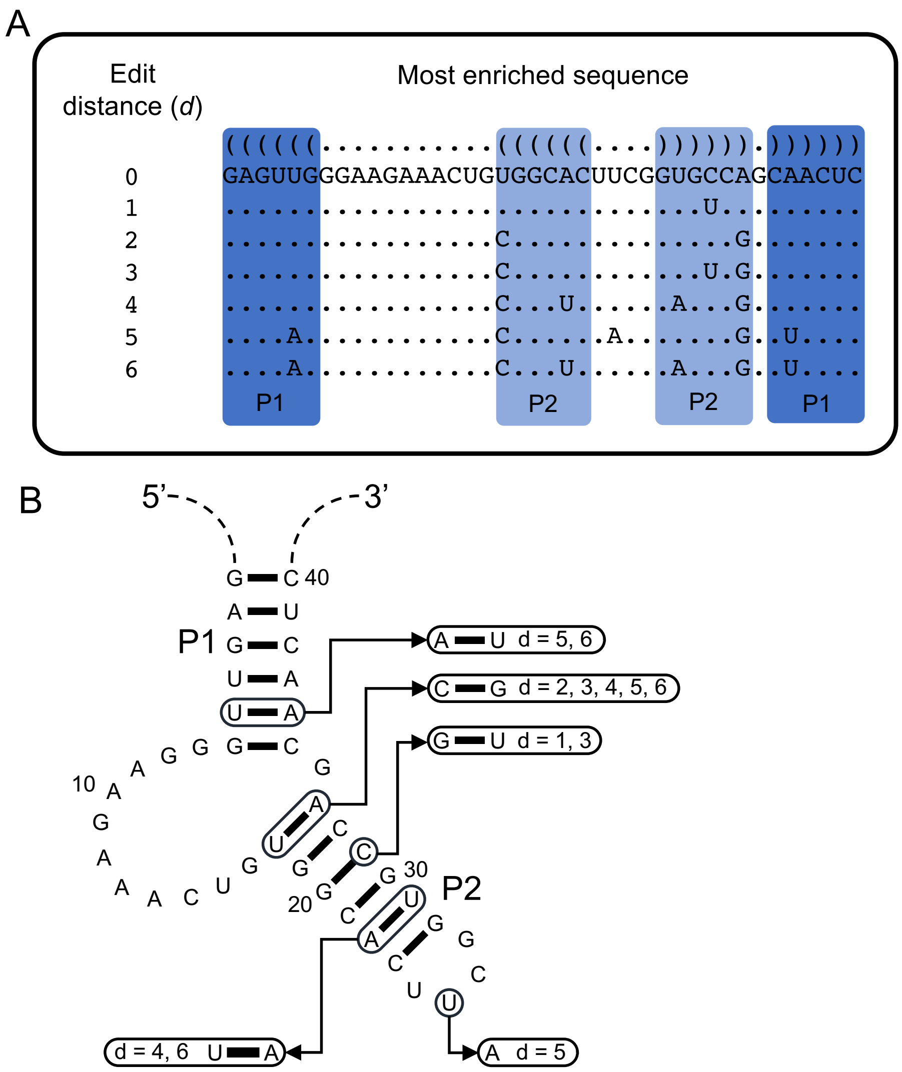

Fig. S2 For the s construct**,** the sequences and structures of the most enriched sequences, at a given edit distance from the "wild-type" sequence, along trajectory 1b. **(A)** Among sequences with a given edit distance (*d*) the most enriched sequence in the population is shown. Sequences are aligned to the "wild-type". Standard bracket notation is used for secondary structures and pairing regions are highlighted and labeled. **(B)** The mutations that are present in the most enriched sequences at a given edit distance are mapped onto the predicted secondary structure, the sites of the mutations are circled within the structure and arrows are used to indicate the altered sequence and any new base-pairs formed.

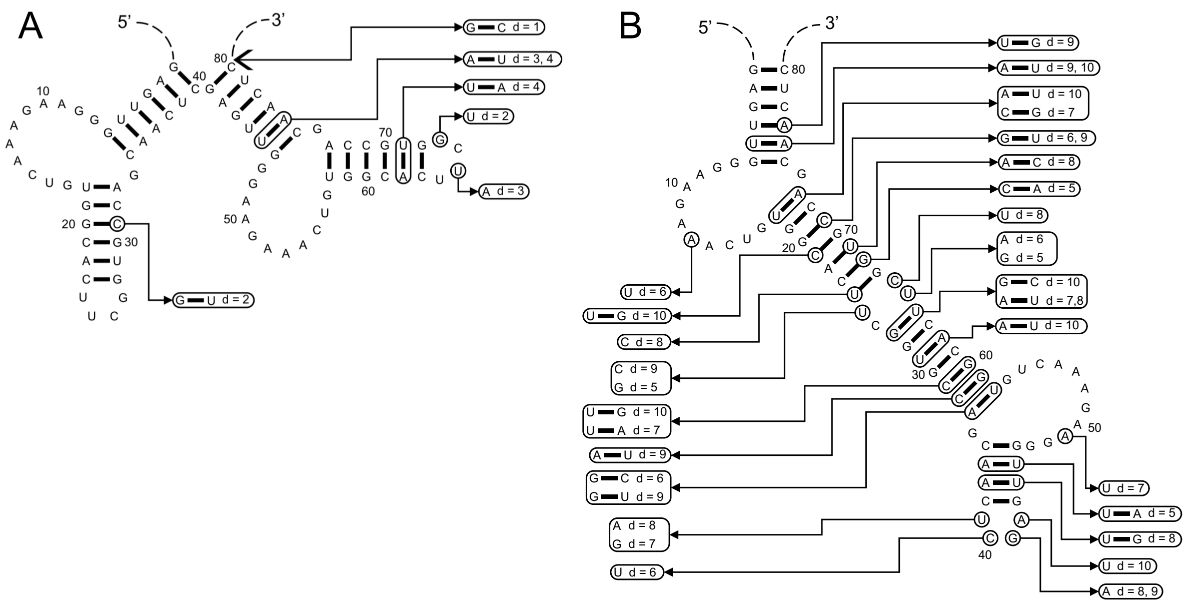

Fig. S3 For the db construct, the mutations from Fig. 5 that are present in the most enriched sequences at a given edit distance are mapped onto their corresponding secondary structures. **(A)** For values of d = 1-4 the mutations are mapped onto the tandem secondary structure, the sites of the mutations circled within the structure and arrows are used to indicate the altered sequence and any new base-pairs formed. **(B)** For values of d = 5-10 the mutations are mapped onto the nested secondary structure.

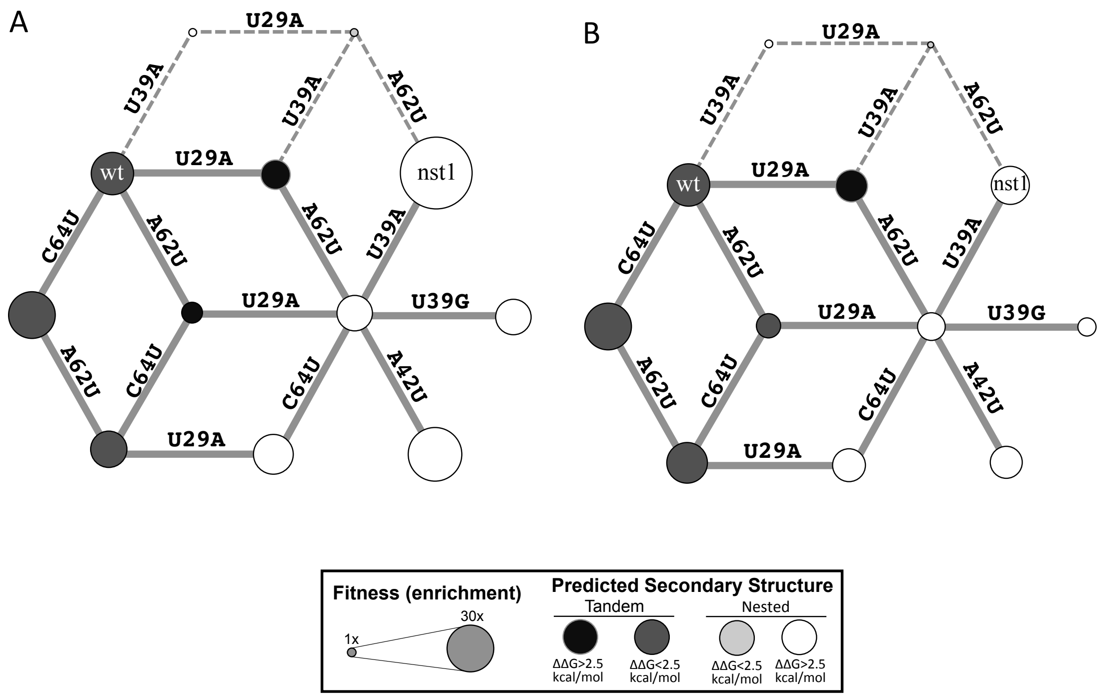

Fig. S4 Additional examples of a connection between tandem and nested structures in sequence space. Sequences are represented by circles and lines connecting them represent single point mutations that convert one sequence to another. Connections to fit sequences are shown as solid lines and connections to sequences that perform poorly in the evolution experiment are shown as dashed lines. The area of the circles is proportional to the enrichment of the sequence along **(A)** trajectory 1b or **(B)** trajectory 1a. The shading of the circles indicates both whether the tandem (black and dark gray) or nested (white and light gray) structure is predicted to be the more stable of the two conformation and the magnitude of the predicted free energy difference between the ensemble of tandem and nested structures. Shading indicates whether the magnitude of the free energy difference is greater than (white and black) or less than (light gray and dark gray)
2.5 kcal/mol.

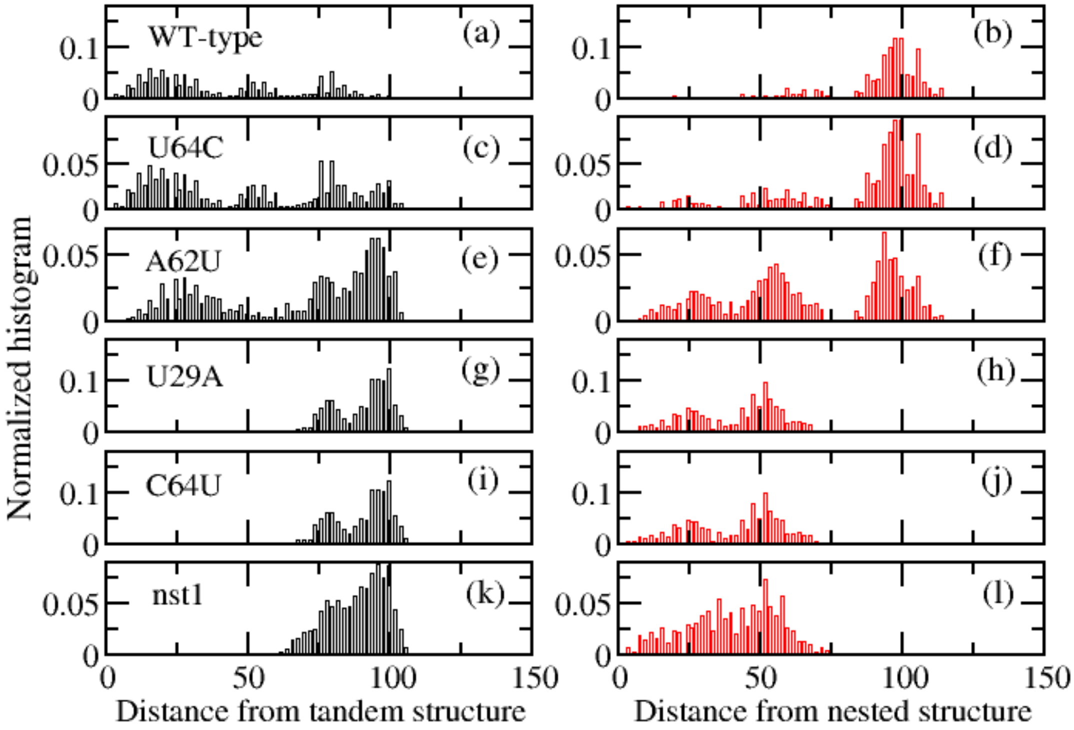

Fig. S5 Histogram for the distance between the predicted suboptimal secondary structures to the tandem (left panel) and nested (right panel) structure, for the six sequences along the shortest evolution path connecting WT-type and nst1 (see Fig. S3). The distance between two secondary structures is calculated with (Hofacker *et al*., 1994) in RNAfold (Lorenz *et al.* 2011).

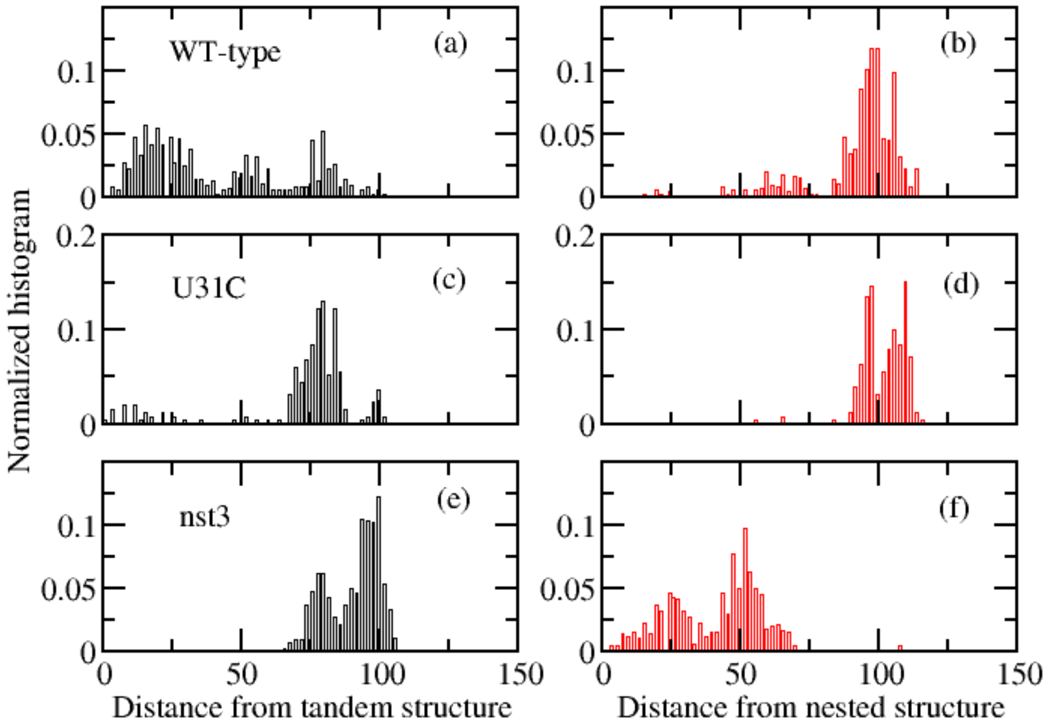

Fig. S6 Histogram for the distance between the predicted suboptimal secondary structures to the tandem (left panel) and nested (right panel) structure, for the three sequences along the shortest evolution path connecting WT-type and nst3 (see Fig. 10). The distance between two secondary structures is calculated with RNAdistance (Hofacker *et al*., 1994) in RNAfold (Lorenz *et al.* 2011).
